# Supplementary material for: Comparing phonological and orthographic networks: A multiplex analysis
Source: PLoS One. 2021 Feb 1;16(2):e0245263. doi: 10.1371/journal.pone.0245263 (PMC7850493; doi:10.1371/journal.pone.0245263)
Supplement: Attachment — Submitted filename: ResponseToReviewers.pdf [file pone.0245263.s002.pdf]

Response to reviewers

Reviewer #1

"The study proposed by the authors in this report is interesting and could yield interesting results. The authors do a good job in motivating their project. The manuscript is well written barring some minor typos (see below). The authors may consider adopting some multilayer community detection algorithms like the ones described in [1] for example to find more interesting clusters across layers.
Typo on line 79 on page 3 - A & B should be V & W.
Reference:
[1] De Bacco, Caterina, et al. "Community detection, link prediction, and layer interdependence in multilayer networks." Physical Review E 95.4 (2017): 042317."

Response: Thank you for your comments. We have added the application of the suggested multilayer community dectection method to the revised version of our Protocol (Lines 129-131), which will enrich our discussion about potential grouping of nodes (words) in the context of the two-layer network. The typo was corrected.

Reviewer #2

1)• Make the networks created with the different distance cutoffs publicly available. Calculating the DL distance is not necessarily trivial and this dataset would be useful to other researchers, who can use it to compare their work or methods to the current ones.
Response: Thank you for your suggestion. We have added the corresponding networks (with 10^4 nodes) for each corpus to our dataset in the Figshare repository. We plan to update the dataset once we have concluded our study.
2) • In the text, define DL as 'Damerau-Levenshtein' before using the acronym.
Response: Corrected in the revised version (Line 23)
3)• I did not identify any connection in the results listed between the phonological and the orthographic networks other than 1 to 1, so I don't think this is rigorously speaking multiplex.
Response: We plan to address the multiplex features in the extended paper
4)• The caption of Fig. 1 states that the illustration is for Spanish but the words are in English in the actual figure.
Response: The error was corrected
5)• Although it is understandable, V should be defined for completeness before Eq. 3.
Response: We have clarified the network notation, and the meaning of the letters (see lines 99-121)
6)• M is used in the definition of the degree of a node right before Eq. 3, but M is never defined. From inspection it seems like M is the multiplex layer, but this should be stated clearly to make it easier for the reader.
Response: We have corrected the description of the network's notation (see lines 99-121)
7)• I did not understand the definition of the modularity (Eq. 6). It says that the partition includes elements C_1, …, C_M. In this case M is subscript. C is defined as the clustering coefficient in Eq. 4. The set S is not really defined. So this needs more work.
Response: Thank you for your comment. In the revised version of the manuscript we have clarified our notation regarding the modularity definition (see Lines 117-121).
8)• The results in Fig. 2 might not be apples to apples because the highest degree depends on the DL distance cutoff. I suggest normalizing the horizontal axis by the highest degree in each network.
Response: We have added the suggested normalization of the horizontal axis in the new version of Fig. 2
9)• I suggest removing Fig. 3 and mentions of specific start dates and due dates. This is not really part of a scientific paper.
Response: The mentioned fig was removed. The new dates were updated in the main text.
